# *Maytenus macrocarpa* (Ruiz & Pav.) Briq.: Phytochemistry and Pharmacological Activity

**DOI:** 10.3390/molecules24122288

**Published:** 2019-06-20

**Authors:** Milan Malaník, Jakub Treml, Veronika Rjašková, Karolina Tížková, Petra Kaucká, Ladislav Kokoška, Peter Kubatka, Karel Šmejkal

**Affiliations:** 1 Department of Natural Drugs, Faculty of Pharmacy, University of Veterinary and Pharmaceutical Sciences Brno, Palackého tř. 1, 61242 Brno, Czech Republic; milan.malanik@seznam.cz (M.M.); v.rjaskova@gmail.com (V.R.); karolina.kalvarova@gmail.com (K.T.); petra.kaucka9@gmail.com (P.K.); 2 Department of Molecular Biology and Pharmaceutical Biotechnology, Faculty of Pharmacy, University of Veterinary and Pharmaceutical Sciences Brno, Palackého tř. 1, 61242 Brno, Czech Republic; tremlj@vfu.cz; 3 Department of Crop Sciences and Agroforestry, Faculty of Tropical AgriSciences, Czech University of Life Sciences Prague, Kamýcká 129, 16500 Praha-Suchdol, Czech Republic; kokoska@ftz.czu.cz; 4 Department of Medical Biology, Jessenius Faculty of Medicine, Comenius University in Bratislava, 03601 Martin, Slovakia; Peter.Kubatka@jfmed.uniba.sk

**Keywords:** dihydro-β-agarofuran sesquiterpene, folk medicine, *Maytenus macrocarpa*, rheumatism, triterpene

## Abstract

*Maytenus macrocarpa* (Celastraceae) is a tree native to Amazonia. Its roots, leaves, bark, and combinations of these are used in traditional medicine mainly to treat rheumatism and, to a lesser extent, to heal wounds and to combat bronchitis and diarrhea. To date, mainly triterpenes and dihydro-β-agarofuran sesquiterpenes were isolated from *M. macrocarpa*. Extracts and selected pure compounds isolated from the leaves, roots, and stem bark showed antibacterial, antiviral, antiparasitic, anti-inflammatory, and cytotoxic activities in vitro. The aim of this review is to summarize the available ethnobotanical, phytochemical, and pharmacological information about this traditional Amazonian medicinal tree, as well as to attract the attention of phytochemists and pharmacognosists to this potentially interesting source of ethnopharmaceuticals.

## 1. Introduction

*Maytenus macrocarpa* (Ruiz & Pav.) Briq. is a tree, up to 30 m tall, belonging to the family Celastraceae. Four hundred different species of the genus *Maytenus* Molina were identified [1]. Although *M. macrocarpa* is the most widely accepted scientific name, *Celastrus macrocarpus* Ruiz & Pav., *Haenkea macrocarpa* (Ruiz & Pav.) Steud., *Haenkea multiflora* Ruiz & Pav., *M. multiflora* (Ruiz & Pav.) Loes., and *M. tarapotensis* Briq. also refer to the same species. It is important to bear in mind that “chuchuhuasha” is a vernacular name for *M. macrocarpa* [2] even though *M. chuchuhuasha* actually refers to another species—*M. krukovii* A.C. Sm. Another used vernacular name, chuchuhuasi, may refer to one or both of the species *M. macrocarpa* and *M. amazonica* Mart. ex Reissek [3]. Other vernacular names in use are chuchuasi, chuchuasha [4], chuchuwasha, chuchuwasha blanca [5], chuchuhuasca [2], and chichtá or xixuá [6].

The fully correct classification of this plant is even more complicated because *M. macrocarpa* is often misidentified as *M. ebenifolia* Reissek or *M. krukovii* A.C. Sm. [1,7]. This chaos raised voices calling for reorganization of this plant family [8]. Recently, the genus *Maytenus* was split into two genera, resurrected *Tricerma* and maintained *Plenckia* and *Fraunhofera* as separate ones. *Maytenus* species in the tropical lineage were transferred to *Monteverdia* [9]; therefore, *Monteverdia macrocarpa* (Ruiz & Pav.) Biral should currently be the scientific name in use instead of *Maytenus macrocarpa*.

A review touching the ethnopharmacology, phytochemistry, and pharmacology of some *Maytenus* species was published [10], but it made no specific reference to *M. macrocarpa*. The database literature search was performed in SciFinder and Google Scholar using keywords “*Maytenus macrocarpa*”, its isolated compounds, and corresponding pharmacological activities.

## 2. Geographical Distribution

The *M. macrocarpa* tree grows exclusively in Amazonia. It is distributed in Bolivia, in Brazil in the state of Acre, in Columbia, in Ecuador in the provinces of Carchi, Esmeraldas, Imbabura, Napo, Pastaza, and Pichincha, in Peru in the regions Amazonas, Huánuco, Loreto, Madre de Dios, Paseo, San Martín, Tumbes, and Ucayali, and in Venezuela in the states of Anzoategui, Apure, Bolivar, and Miranda [1,2,4,6]. It grows mainly in lowland tropical rainforests, but exceptionally can be found up to 2000 m above sea level [1].

## 3. Phytochemistry

*M. macrocarpa* was used in tribal folk medicine for many years, and this aroused interest in the compounds it contains. We searched the available literature for phytochemical research carried out on substances presented in *M. macrocarpa*. As previously mentioned, the situation is complicated by the fact that several synonymous names are used for this plant, and the literature describing its phytochemical analysis is not very rich.

Mejia and Rengifo (1995) reported that *M. macrocarpa* contains some simple phenols and quinones, but this reference lacks exact descriptions of the structures identified [4]. The triterpenoids are a much better explored group of compounds isolated from *M. macrocarpa*. Several studies described the presence of mainly tetracyclic dammarane and pentacyclic friedelane triterpenes, and to a lesser extent quinonmethide, lupane, and oleane derived compounds. Another very interesting group of compounds obtained from *M. macrocarpa* consists of dihydro-β-agarofuran sesquiterpenes. Table 1, including the corresponding references, presents a full list of the compounds isolated to date. Compounds presented in the Table 1 belong to various groups: dihydro-β-agarofuran sesquiterpenes (**1**–**3**), dammarane triterpenes (**4**–**13**), lupane triterpenes (**14**–**18**), and pentacyclic triterpenes (**19**–**45**).

## 4. Folk Medicine

The *M. macrocarpa* plant is very popular in South American folk medicine. *M. macrocarpa* preparations are used to treat rheumatism almost everywhere in Amazonia. Other uses vary in the different regions. Ethnopharmacological studies showed that *M. macrocarpa* possesses aphrodisiac and anti-diarrheic effects and can also be used as a health and postpartum tonic and to enhance the healing of broken bones [5]. It has antimalarial [3,25] and antileishmanial effects [3]. Some local tribes use it to treat unspecified colds and women’s ailments, to enhance wound healing [26], or to cure skin cancer [27].

The most common local formulations use the stem bark and root of *M. macrocarpa* either decocted or macerated in the local rum, which is prepared from sugar cane [5]. The following traditional recipes are presented here to illustrate the situation: for the treatment of rheumatism, macerate 250 g of dried shredded roots in alcohol and drink a glass on an empty stomach in the morning and evening regularly for a period of one month. The stem bark of *M. macrocarpa* can be used for the same purpose. For treatment of cold and bronchitis, boil 200 g of stem bark in 2 L of water for 1 h, add 250 mL of local rum, and allow this to macerate for ten days. Then, swallow a spoonful of this remedy every morning for 15 days [4]. Another interesting use of *M. macrocarpa* is as a component remedy of a strict diet that is part of a ritual procedure called *sama*, used by locals in the Chazuta valley of Peru. The *sama* is a 2–8-week-long period of fasting during which plants with laxative and emetic effects are primarily consumed, supplemented with plants with other effects, e.g., anti-rheumatic effects [28].

## 5. Pharmacological Activities

### 5.1. Antibacterial and Antifungal Activity

The increasing resistance of bacteria to antibiotics that are currently in use is forcing scientists to seek new compounds with strong antimicrobial activity. It is difficult to make a meaningful comparison of the results obtained in different antibacterial tests because the extracts and panels of test organisms employed are not standardized and different growth media were used in the assays. Some general endpoint criteria for assigning the activities of compounds or extracts, e.g., the half maximal inhibitory concentration (IC_50_) of the antibacterial effect below 100 µg/mL for test extracts and the IC_50_ below 25 µM for pure compounds were adopted, but the close values obtained by testing the extracts are accepted more readily. Some scientific teams use assays that generate results expressed in minimal inhibitory concentration (MIC) or minimal bactericidal concentration (MBC), which can be tricky to compare. Furthermore, the minimal requirement for valid antibacterial assays is the use of at least one strain of Gram-positive and one strain of Gram-negative bacterium [29]. According to a study conducted by Kloucek et al., an ethanolic extract obtained from the root bark of *M. macrocarpa* showed activity against *Bacillus cereus* American Type Culture Collection (ATCC) 11778, *Bacillus subtilis* ATCC 6633, *Staphylococcus epidermidis* ATCC 12228, *Streptococcus pyogenes* ATCC 19615, *Escherichia coli* ATCC 25922 (all at the MIC 125 µg/mL), and *Enterococcus faecalis* ATCC 29212, *Staphylococcus aureus* ATCC 25923, *Bacteroides fragilis* ATCC 25285, *Pseudomonas aeruginosa* ATCC 27853, and *Candida albicans* ATCC 10231 (all at the MIC of 250 µg/mL), whereas an ethanolic extract of stem bark did not show any activity against the same panel of bacterial strains [30]. The differences between the results of the antibacterial activity testing of extracts prepared from roots and those from stem bark suggest that the spectrum of compounds present in root parts is different from the compounds present in aerial parts. The active extracts showed activity comparable to the antibiotic (ciprofloxacin) used as the standard control [31]. However, the bacterial suspensions at a density of 10^7^ colony-forming units (CFU)/mL used for the tests could cause false-negative results [29], as confirmed by the data obtained for some compounds isolated from or present in *M. macrocarpa*. Scutione (**24**) showed strong activity against 11 Gram-positive bacterial strains (MIC 0.1–2.0 µg/mL), but no activity against eight Gram-negative bacterial strains (Gonzalez et al. 1996). In the same study, scutione (**24**) showed modest cytotoxic activity against HeLa, Hep-2, and Vero cell lines. It would, therefore, be worth testing scutione (**24**) against methicillin-resistant *S. aureus* (MRSA) and vancomycin-resistant *Enterococci* (VRE) because of the threat they pose to the human population.

The betuline derivative 3-(*Z*)-*cis*-coumaroylbetulin (**15**) showed relatively good activity against *P. aeruginosa* and *S. aureus* at a concentration of 0.1 mg/disc, whereas its *trans* isomer (**16**) showed much less activity. The antimicrobial activity was evaluated by the paper disc–agar diffusion method, where each disc (6 mm) was aseptically impregnated with 10 μL of the solution of the test compound [32]. Maytenfolic acid (**38**) was shown to be active against *S. aureus* (MIC = 12.5 μg/mL) and *P. aeruginosa* (MIC = 12.5 μg/mL) [33]. Neither 24-(*Z*)-3-oxo-dammara-20(21),24-dien-27-oic acid (**13**) nor octa-*nor*-13-hydroxydammara-1-en-3,17-dione (**45**) exhibited toxicity for any of the eight yeast strains tested using concentrations of at least 100 µg/mL [12]. Friedelin (**19**) was identified as an antimycobacterial compound against *Mycobacterium madagascariense* and *M. indicus pranii* [34], but it showed low antimycobacterial activity against *M. tuberculosis* [35] and, similarly to canophyllol (**20**) [36], little or no activity against several fungal and bacterial species [37,38,39,40,41]. The growth of several bacterial species was inhibited by friedelin (**19**) according to the work of Viswanathan et al. [42], Ragasa et al. [43], Jain et al. [44], and Singh and Dubey [45]; the good antibacterial activity of this compound against both Gram-positive and Gram-negative bacterial strains was described by Kuete et al. [46,47] and Sahiq Ali et al. [48]; its weak activity against *S. aureus* was observed in a study done by Chiozem et al. [49].

### 5.2. Antiviral Activity

Triterpenes, especially pentacyclic triterpenes and their derivatives, are known for their antiretroviral activity [50,51]. Betulinic acid and its derivatives were especially well explored for this effect [52]. Studies reported that pentacyclic triterpenes inhibit human immunodeficiency virus (HIV) reverse transcriptase [53]. Several pentacyclic triterpenes were isolated from *M. macrocarpa*. Piacente et al. [13] conducted an assay with a panel of 13 pentacyclic triterpenes isolated from a chloroform extract of the bark of *M. macrocarpa*: triptotriterpenonic acid A (**39**), 3-(*E*)-caffeoylbetulin (**14**), macrocarpoic acid A (**36**), macrocarpoic acid B (**37**), maytenfolic acid (**38**), macrocarpol A (**43**), 3-(*E*)-caffeoyluvaol (**44**), 3-(*Z*)-*p*-coumaroylbetulin (**15**), nepeticin (**17**), orthosphenic acid (**42**), 22-*epi*-maytenfolic acid (**40**), 22-*epi*-triptotriterpenonic acid A (**41**), and 3-(*E*)-*p*-coumaroylbetulin (**16**). These compounds were tested for anti-HIV activity in C8166 T cells infected with the HIV-1MN strain. The most effective compound was shown to be 22-*epi*-triptotriterpenonic acid A (**41**), which inhibited the interaction between the glycoprotein gp120 located on the HIV envelope and the cluster of differentiation 4 (CD4) receptor of T cells by 55% at the half maximal effective concentration (EC_50_) = 1 µg/mL. The selectivity index (the ratio of the cytotoxicity value against the particular cell line used to test the observed antiviral effect) was found to be 35, which is sufficient to warrant further testing [13]. However, it is difficult to compare results obtained from this study with other studies reporting the antiviral activities of other triterpenes because different research groups used different viral strains and infected cells, and, in many cases, the data for positive controls were not published. Testing of further compounds showed that triptotriterpenonic acid A (**39**) is also active as an antiviral compound, but it is not selective enough when compared with substances currently used in clinics [13]. Furthermore, 22-*epi*-maytenfolic acid (**40**) inhibited HIV replication in H9 lymphocyte cells with an EC_50_ value of 5.65 µg/mL [54]. Investigation of lupane derivatives revealed nepeticin (**17**) as a moderate inhibitor of HIV-1 replication in MT-2 cells infected with an X4 tropic HIV (NL4.3-Ren) (IC_50_ = 10.4 µM), whereas 3-(*E*)-caffeoylbetulin (**14**) and lupeol (**18**) were inactive [55].

The anti-HIV replication activities in H9 lymphocyte cells of friedelin (**19**) and canophyllol (**20**) were shown to be greater than 10 μg/mL [56]. However, friedelin (**19**) was found to have no activity when tested against hepatitis virus type C [57], it showed no HIV RNAase inhibitory activity [58], and also did not affect reverse transcriptase [59,60]. Low activity of friedelin (**19**) against para-influenza virus type3 was also observed in a study by Jiang et al. [61].

### 5.3. Antiparasitic Activity

Malaria kills more than two million people every year. Its etiological agent *Plasmodium* spp. frequently shows resistance to chloroquine and, for this reason, many scientists focused their research on finding new antimalarial remedies. Plant remedies would be a relatively convenient solution because of their accessibility [25]. An ethanolic extract from the bark of *M. macrocarpa* was tested for antimalarial and antileishmanial activities in ethnopharmacological studies carried out in Loreto, Peru [3]. It inhibited a chloroquine-susceptible strain of *P. falciparum* 3D7 at doses lower than 10 µg/mL (toxicity to human blood lymphocytes was proven at a dose of 48 µg/mL) in vitro [3]. Recently, Vásquez-Ocmín et al. reported a study of 50 extracts from 46 medicinal plants used traditionally against protozoan diseases in Loreto (Peru) and an ethanolic extract from the bark of *M. macrocarpa* was one of the most active against *P. falciparum* 3D7 chloroquine-sensitive strain and against *P. falciparum* W2 chloroquine-resistant strain with IC_50_ = 0.02 µg/mL for both strains [62]. Pristimerin (**26**), an active compound isolated from *M. macrocarpa*, is unfortunately toxic to human cells (HT-29) [3,63], but only at concentrations approximately 10 times higher than required to kill *P. falciparum* K1 and *P. falciparum* NF54 (IC_50_ 190.4 and 270.9 ng/mL, respectively) [63]. Other studies showed that 3-(*E*)-caffeoylbetulin (**14**) was inactive at >50.0 μg/mL against *P. falciparum* K1 (multidrug-resistant strain) [64], and 3-(*Z*)-*p*-coumaroylbetulin (**15**) and 3-(*E*)-*p*-coumaroylbetulin (**16**) possess only low activity when tested on mice infected with *P. berghei* [65]. Friedelin (**19**) and canophyllol (**20**) showed some antiplasmodial activity when tested on the *P. falciparum* W2 strain (resistant to chloroquine) with IC_50_ values of 7.2 and 15.0 μM, respectively [66]; lower activity was shown in a study by Mitaine-Offer et al. [67], when the activities of friedelin (**19**) and canophyllol (**20**) were compared to chloroquine (on both chloroquine-resistant and chloroquine-sensitive *P. falciparum* strains). Although an ethanolic extract from the bark of *M. macrocarpa* was active against both *P. falciparum* 3D7 and *P. falciparum* W2, reports of any antimalarial-active compounds found in *M. macrocarpa* are relatively scarce. According to Cos et al. [29], research on potentially antimalarial-active compounds obtained from *M. macrocarpa* would benefit from the use of drug-resistant strains of *Plasmodium*.

Leishmaniasis is a serious disease that affects the developing world in particular. Three different forms of this disease are described—a cutaneous form (the most common), a mucocutaneous form (leading to partial or total destruction of mucous membranes), and a visceral form (the most serious form that can be lethal). Ethnopharmacological studies of *M. macrocarpa* describe its use to treat different wounds. Could the “wounds” reported by local people be the cutaneous form of leishmaniasis? The *Leishmania major* strain (the World Health Organization (WHO) referential strain) used in a brief antileishmanial-activity screening of an extract obtained from *M. macrocarpa* bark showed relatively strong inhibitory activity at doses lower than 10 µg/mL. Unfortunately, no strains of local clinically isolated *Leishmania* parasites were available for this study, and only the promastigote form of *Leishmania* was used in the assay. The predictive value of the test is, therefore, limited, and more experiments should be carried out to confirm the effect [3]. A large study of the molecular docking of different substances, including a series of triterpenic compounds present in *M. macrocarpa*, identified the molecular protein targets of several *Leishmania* species [68]. Friedelin (**19**) preferentially targeted *L. major* tyrosyl-tRNA synthetase (docking energy = −102.4 kJ/mol), whereas pristimerin (**26**) was strongly docked to *L. major N*-myristoyltransferase (docking energy = −112.9 kJ/mol), and lupeol (**18**) exerted the lowest-energy docked pose with *L. major* nucleoside hydrolase (docking energy = −99.0 kJ/mol) [68]. Despite promising in silico results, friedelin (**19**) and epifriedelinol (**29**) showed no significant activity against *L. donovani* promastigotes [69,70]. Experiments with 3-caffeoylbetulin (**14**) showed it to be inactive against *L. major* as well [71]. Additionally, 1α,6β,8β,15-tetraacetoxy-9α-benzoyloxy-4β-hydroxy-β-dihydroagarofuran (**2**), a dihydro-β-agarofuran sesquiterpene, is a promising antileishmanial substance isolated from *M. macrocarpa*. Alone, it possesses little direct cytotoxic activity against *L. tropica*, but it can also contribute to the effect of the currently used drug daunomycin by inhibiting the P-glycoprotein pump. This ATP-dependent transporting protein causes the efflux of daunomycin from cells [72]. Dihydro-β-agarofuran sesquiterpenes block the P-glycoprotein pump and more daunomycin stays inside the cells to kill *Leishmania* more effectively. Blockers with greater affinity were, however, already discovered [73].

An in silico molecular docking study was performed to investigate potential bioactive substances of antitrypanosomal plants. Although friedelin (**19**) was relatively weak docking ligands, it docked selectively with *T. brucei* uridine diphosphate (UDP)-galactose 4’-epimerase [74]. On the other hand, friedelin (**19**) showed no antitrypanosomal activity in vitro [70,75,76].

### 5.4. Cytotoxic Activity

Many studies of the cytotoxic activity of plant secondary metabolites were carried out, because of the urgent need for new remedies to treat cancer. *M. macrocarpa* was also examined as a potential source of cytotoxic substances. The direct cytotoxicity and mechanisms of effect of several compounds isolated from *Maytenus* were both analyzed. Pristimerin (**26**) inhibited DNA synthesis and triggered apoptosis in human HL-60 cells (promyelocytic leukemia cell line). It inhibited topoisomerase II, but it did not influence topoisomerase I [19]. Pristimerin (**26**) also showed activity against the following cell lines with IC_50_ values ranging from 0.55 µM to 3.20 µM: K-562 (chronic myelocytic leukemia), SF-295 (glyoblastoma), HCT-8 (colon carcinoma), and MDA/MB-435 (melanoma). However, except for MDA/MB-435, the dosage needed for the IC_50_ effect was greater than that for doxorubicin, which is taken as the standard reference drug. Pristimerin (**26**) was toxic to PBMC (peripheral blood mononuclear cells) in the same concentration range, suggesting that this compound is relatively unselective [19]. Another assay showed the cytotoxic potential of pristimerin (**26**) against HL-60 (IC_50_ = 0.2 µM) and MCF7 (breast adenocarcinoma) (IC_50_ = 0.4 µM) cell lines [77]. Recently, the NCI-60 cell line screen revealed that pristimerin (**26**) was active against UO-31 (renal carcinoma), T-47D (breast cancer), and A549 (non-small-cell lung cancer) human tumor cell lines with individual half maximal inhibition of cell proliferation (GI_50_) values ranging from 0.12 µM to 1.2 µM [78]; therefore, pristimerin (**26**) would deserve further in vivo evaluation. Macrocarpine A (**32**), macrocarpine B (**33**), macrocarpine C (**34**), and macrocarpine D (**35**) were shown to be active against P-388D1 (mouse lymphoma), A-549 (human lung carcinoma), HT-29 (human colon carcinoma), and MEL-28 (human melanoma) cells with IC_50_ ranging between 0.4 and 5.2 µM [24], whereas 28-hydroxyfriedelane-1,3-dione (**22**) was inactive against the same panel of cancer cell lines [16], but a positive control was missing in both studies. Compounds macrocarpine A–D (**32–35**) were isolated from a hexane fraction, which shows the relatively lipophilic characteristic that could impede their solubility in water and, therefore, the likelihood that they could be used as drugs [24]. An assay by Oramas-Royo et al. showed the activity of macrocarpine A (**32**) against HL-60 cells (IC_50_ = 1.7 µM) [77]. Vitideasin (**25**) was active against six solid tumor cell lines with IC_50_ ranging between 2.7 and 5.4 µM [79]. However, only in vitro studies are presented so far, and in vivo studies are needed. Recently, a review showing the anti-cancer potential of celastrol (**28**) was published [80].

Derivatives of betulin are well known for cytotoxic properties. Several of them were isolated from *M. macrocarpa* (**14–18**). Furthermore, 3-(*E*)-caffeoylbetulin (**14**) and 3-(*Z*)-*p*-coumaroylbetulin (**15**) showed antitumor-promoting properties when tested in assays using the inhibition of soft agar colony induction by 12-*O*-tetradecanoylphorbol-13-acetate (TPA) in JB6 cells [81]. Similarly, 3-(*E*)-caffeoylbetulin (**14**) was reported as a potent cytotoxic agent against SK-OV-3 (ovary malignant ascites) and SK-MEL-2 (skin melanoma) human cancer cell lines with IC_50_ values of 9.0 and 2.9 µM, respectively [82]. In another study, 3-(*E*)-caffeoylbetulin (**14**) exhibited moderate cytotoxic activity against KB (human oral epidermoid carcinoma) and NCI-H187 (human small-cell lung cancer) cell lines with IC_50_ values of 28.4 and 16.2 µM, respectively. Unfortunately, 3-(*E*)-caffeoylbetulin (**14**) was cytotoxic also against Vero cells (IC_50_ = 8.9 µM) [64]. On the other hand, a different study of 3-(*E*)-caffeoylbetulin (**14**) showed no significant cytotoxicity against KB/S, KB/VJ300, and KU 19-20 cells [83]. Additionally, 3-(*E*)-*p*-coumaroylbetulin (**16**) showed activity against KB and HUVEC (human umbilical vein endothelial cells) cell lines [84]. Scutione (**24**) was tested for cytotoxic activity on HeLa (IC_50_ = 4.9 µg/mL), Hep-2 (IC_50_ = 5.6 µg/mL), and Vero (IC_50_ = 7.2 µg/mL) cell lines and was not proven to be active compared with a positive control. This suggests dose-dependent activity against bacterial and mammalian cells and discriminating, non-specific toxicity [18]. Canophyllol (**20**) and friedelin (**19**) showed moderate activity against HL-60 cells with IC_50_ values of 17.1 and 48.5 µM, respectively [85]. In addition, friedelin (**19**) was used as a lead compound for the synthesis of several analogs with a dose-dependent ability to inhibit the catalytic activity of human topoisomerase IIα [86]. The activity of friedelin (**19**) was found to be responsible for the anti-tumor effect of bamboo shavings used in traditional Chinese medicine [87]. Experiments using incubation with HeLa cells showed that the IC_50_ of friedelin (**19**) was 37 μM. Friedelane-type triterpenoids also showed some anti-tumor promoting activity. Friedelin (**19**) had an inhibitory effect on Epstein–Barr virus early antigen (EBV-EA) activation induced by TPA [88]. The application of selected triterpenes reduced inflammation in mouse ears induced by TPA and inhibited tumor genesis [89]. A search in literature for the bioactivity of lupeol (**18**) found a review showing it has anti-cancer potential [90].

Sesquiterpenes isolated from the leaves of *M. macrocarpa*, such as 6β,8β,15-triacetoxy-1α,9α-dibenzoyloxy-4β-hydroxy-β-dihydroagarofuran (**1**) and 1α,6β,8β,15-tetraacetoxy-9α-(benzoyloxy)-4β-hydroxy-β-dihydroagarofuran (**2**), were tested against P-388D1, A-549, HT-29, and MEL-28 cancer cell lines, but showed no significant cytotoxic activity [11].

### 5.5. Anti-Inflammatory Activity

The focus on anti-inflammatory activity was emphasized on the basis of usage of *M. macrocarpa* as a component of medicinal preparations for treating rheumatism, which was mentioned by almost all of the local people who were questioned during the ethnopharmacological research in the area of the natural distribution of the tree, irrespective of the geographic region or ethnic group [5,26].

Celastrol (**28**), a pentacyclic triterpene isolated from *M. macrocarpa*, was tested for anti-inflammatory activity. This compound is also connected to Chinese medicine, where it is isolated from the medicinal plant *Tripterygium wilfordii* Hook (Celastraceae) and used to treat rheumatoid arthritis and spondylitis [91]. Celastrol (**28**) was successful in many in vitro assays. It inhibited the release of interleukin (IL)-1α and IL-1β from lipopolysaccharide-stimulated human PBMC cells, inhibited activation of nuclear factor kappa B (NF-κB) and caspase-1, and reduced the secretion of IL-1β and tumor necrosis factor (TNF)-α in a human THP-1 macrophage-like cell line, and already went through in vivo tests in rats as an effective blocker of IL-1β and TNF-α, two cytokines connected with the development and progression of rheumatoid arthritis [91,92]. The question arises as to whether celastrol (**28**) is the only compound contained in *M. macrocarpa* that is responsible for anti-inflammatory activity. Some sources show that it may not be like that. In fact, 3-(*E*)-caffeoylbetulin (**14**) inhibited nitric oxide (NO) production (9.3 ± 3.2% of inhibition at 10 μM) and the formation of prostaglandin E2 (PGE2) (IC_50_ = 10.8 μM) [93], and 24-(*E*)-3-oxo-dammara-20,24-dien-26-ol (6) displayed moderate NO inhibitory activity (IC_50_ = 22.36 μM) [94] when tested in vitro on a system of RAW 264.7 cells stimulated by lipopolysaccharide (LPS). Lupeol (**18**) is a well-known substance with a multi-target anti-inflammatory potential as reported in reviews by Wal et al. [95] and Siddique and Saleem [96]. Oliveira-Junior et al. very recently demonstrated that lupeol (**18**) (0.1 μM) exhibited anti-neuroinflammatory and neuroprotective activity in cerebellar cells [97]. Friedelin (**19**) can also possibly contribute to anti-inflammatory action [98], as shown by its lipoxygenase inhibitory activity [99] and several in vivo assays on mice [100]. On the other hand, cyclooxygenase was inhibited only slightly and lipoxygenase not at all in another study [101], and no inhibition of the platelet-activating factor (PAF)-stimulated release of β-glucuronidase from polymorphonuclear leukocytes was observed [102]. Friedelin (**19**) was identified as an active substance in a test of the inhibition of carrageenan-induced paw edema in rats [103], but an in vivo assay showed no activity of friedelin (**19**) and epifriedelinol (**29**) in the indomethacin-induced ulcer model in rats [104]. Friedelin (**19**) only slightly inhibited the production of NO in RAW 264.7 cells [105], and did not greatly inhibit the activity of nitric oxide synthase (NOS) in murine microglial cells [106], but it moderately inhibited the secretion of TNF-α in the latter cell line at the relatively low concentration of 100 nM [107]. Little activity was observed when friedelin (**19**) was tested for the inhibition of human leukocyte elastase (68% at a concentration of 25 μg/mL), which was quite different from canophyllol (**20**), which showed much greater activity (IC_50_ = 2.5 μM) [108]. Additionally, canophyllol (**20**) induced secretion of cytokines IL-6, IL-12, and TNF-α in PBMC cells [109], and triptotriterpenonic acid A (**39**) showed a weak inhibitory effect on IL-2 release (27.0% inhibition at 10 μg/mL) and interferon (IFN)-γ release (66.7% inhibition at 10 μg/mL) produced by lipopolysaccharide-stimulated human PBMC cells [21].

### 5.6. Other

Among other pharmacological activities described in the literature we found a positive effect of compounds from *M. macrocarpa* on impaired metabolic conditions, such as diabetes mellitus and obesity. Additionally, 3-(*E*)-caffeoylbetulin (**14**) showed weak α-glucosidase inhibitory activity (10.6% inhibition at 10 μg/mL) [110], while 1α,6β,8β,15-tetraacetoxy-9α-(benzoyloxy)-4β-hydroxy-β-dihydroagarofuran (**2**) displayed good α-glucosidase inhibitory activity with IC_50_ values of 42.58 μM [111]. Very recently, in vivo, in vitro, and in silico studies reported that lupeol (**18**) possesses antidiabetic effects through a peroxisome proliferator-activated receptor (PPAR)δ/γ dual agonist action [112], and canophyllol (**20**) stimulates the expression and translocation of glucose transporter 4 (GLUT4) in L6 myotubes in vitro (30 μg/mL), as well as in vivo in insulin-sensitive tissues of KK-Ay mice (60 mg/kg/day) via activation of the AMP-activated protein kinase (AMPK) pathway [113].

On the other hand, 22-*epi*-maytenfolic acid (**40**) (IC_50_ = 26 μM) and maytenfolic acid (**38**) (IC_50_ = 72 μM) show inhibitory effects on rat lens aldose reductase, a key enzyme in the polyol pathway, where it catalyzes the reduction of glucose to sorbitol. Sorbitol does not readily diffuse across cell membranes, and the cellular accumulation of sorbitol was implicated in chronic complications of diabetes, such as cataracts [114].

Maytenfolic acid (**38**) was shown to induce lipolysis in rat epididymal fat-derived adipocytes at 100 mg/L (100 μg/mL) [115]. The role of celastrol (**28**) in metabolic diseases was well reviewed by Lan et al. [116].

### 5.7. Toxicity Studies

Only one in vivo study using an ethanolic extract obtained from leaves of *M. macrocarpa* and intended to assess the safety of *M. macrocarpa* preparations was reported to date. A negative inotropic effect on the heart rate at a dose of 1500 mg/kg (1500 μg/g) was observed. Furthermore, no significant changes in the rectal temperature of rats were recorded at doses of 500 mg/kg (500 μg/g), 1000 mg/kg (1000 μg/g), and 1500 mg/kg (1500 μg/g) [117]. However, the extract was administrated to the rats intraperitoneally, which is not the usual method of application. Although we found many inaccuracies in this research, we can assume that this drug should be safe at the dosages traditionally used, but more research would be welcome and reassuring.

## 6. Conclusions

This review was prepared to summarize the ethnobotanical, phytochemical, and pharmacological information about *M. macrocarpa*, a tree in the Celastraceae family. It is distributed in tropical lowland rainforests, with some exceptions growing up to 2000 m above sea level. Only Kvist et al. mentioned the possibility of different constituent compounds occurring at different altitudes, but even these authors suggested this could be due to confusion involving the vernacular name chuchuhuasi, which may refer to one or both of the species, *M. macrocarpa* and *M. amazonica*, with the former found mostly in flood-plain forests and the latter in upland forests [3]. Examination of previous studies shows clearly that no differences between the content of compounds of lowland and highland trees were reported. Root and stem bark preparations of *M. macrocarpa* are widely used in Amazonian folk medicine to treat rheumatism and parasitic diseases. They are so popular that harvesting depleted the local *M. macrocarpa* trees close to villages [26]. Even so, the evidence needed to register it as an evidence-based drug is insufficient. The bioactive substances must be identified, and in vivo studies of their effects must be carried out. The best explored compounds of *M. macrocarpa* are tetracyclic and pentacyclic triterpenes, and the dihydro-β-agarofuran sesquiterpenes are also noteworthy. Unfortunately, most of the compounds that were discovered are relatively large lipophilic molecules, and their resultant limited solubility in water makes it harder to use them as drugs. Nevertheless, progress in pharmaceutical technology (e.g., nanocarriers, encapsulation) could improve their bioavailability and solve this problem.

Summarizing the biological tests, the vast majority of assays were carried out in vitro, and they are yet to bring forth any promising active compounds. Despite promising results of antibacterial activity of an ethanolic extract obtained from the root bark of *M. macrocarpa*, the antibacterial activity of isolated compounds is not very strong compared to currently used antibiotics. Only scutione (**24**) displayed strong antibacterial activity and no cytotoxicity; it would, therefore, be worth testing against methicillin-resistant *S. aureus* (MRSA) and vancomycin-resistant *Enterococci* (VRE) because of the urgent medical need for new antibiotics effective against these highly aggressive and resistant bacterial strains.

Pharmacokinetic and toxicological studies still do not exist, but it can be deduced from traditionally used doses of long-term local preparations that *M. macrocarpa* is relatively safe. More studies should be carried out to determine the full medicinal value of *M. macrocarpa*. No events of deaths or serious health problems resulting from the use of extracts obtained from *M. macrocarpa* were reported; therefore, we suppose it is safe to ingest.

## Figures and Tables

**Table 1:** Compounds isolated from *Maytenus macrocarpa*.

Class	Name of Compound	Structure				Plant Part	Reference
**Dihydro-β-agarofurane sesquiterpene**	6β,8β,15-triacetoxy-1α,9α-dibenzoyloxy-4β-hydroxy-β-dihydroagarofuran (**1**) 1α,6β,8β,15-tetraacetoxy-9α-(benzoyloxy)-4β-hydroxy-β-dihydroagarofuran (**2**) (1*S*,4*S*,6*R*,7*S*,8*S*,9*R*)-1,6,15,triacetoxy-8α,9β-dibenzoyloxy)-4β-hydroxy-β-dihydroagarofuran (**3**)	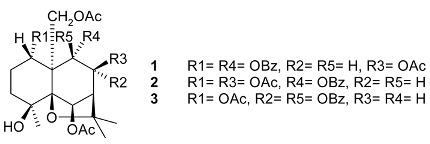				**Leaves**	[11]
**Dammarane triterpenes**		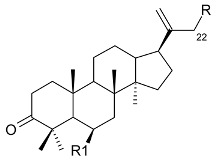					
		**R**		**R1**			
	24-(*E*)-3-oxo-dammara-20,24-dien-26-al (**4**) 24-(*Z*)-3-oxo-dammara-20,24-dien-26-al (**5**)	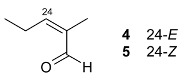		H		Stem bark exudate	[11,12]
	24-(*E*)-3-oxo-dammara-20,24-dien-26-ol (**6**)	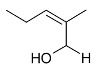		H			
	24-(*E*)-3-oxo-dammara-23α-hydroxy-20,24-dien-26-al (**7**) 24-(*E*)-3-oxo-dammara-23β-hydroxy-20,24-dien-26-al (**8**)	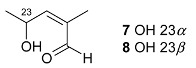		H			
	24-(*E*)-3-oxo-dammara-6β-hydroxy-20,24-dien-26-al (**9**)	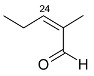		OH			
	24-(*E*)-3-oxo-dammara-6β-hydroxy-20,24-dien-26-ol (**10**)	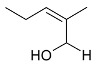		OH			
	23-(*Z*)-3,25-dioxo-25-*nor*-dammara-20,24-dien (**11**)	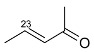		H			
	24-(*E*)-3-oxo-22-hydroxy-23-methylene-dammara-20,24-dien-26-oic acid (**12**)	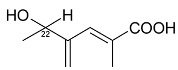		H			
	24-(*Z*)-3-oxo-dammara-20(21),24-dien-27-oic acid (**13**)	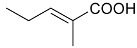		H			
**Lupane triterpenes**		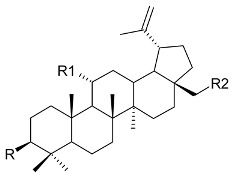					
		**R**		**R1**	**R2**	Bark	
	3-(*E*)-caffeoylbetulin (**14**)	3-(*E*)-caffeoyl		H	OH		[13]
	3-(*Z*)-*p*-coumaroylbetulin (**15**)	3-(*Z*)-*p*-coumaroyl		H	OH		
	3-(*E*)-*p*-coumaroylbetulin (**16**)	3-(*E*)-*p*-coumaroyl		H	OH		
	nepeticin (**17**)	OH		OH	H		
	lupeol (**18**)	OH		H	H		[11]
**Pentacyclic triterpenes**		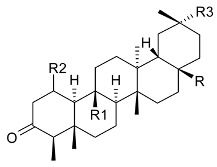					
		**R**	**R1**	**R2**	**R3**		
	friedelin (**19**)	CH_3_	CH_3_	H	CH_3_	Stem bark exudate, leaves	[12,14,15,16]
	canophyllol (**20**)	CH_2_OH	CH_3_	H	H	Stem bark exudate	[16,17]
	3-oxofriedelan-25-al (**21**)	CH_3_	CHO	H	H		[12,16,17]
	28-hydroxyfriedelane-1,3-dione (**22**)	CH_2_OH	CH_3_	=O	H		[12,16]
	3-oxo-29-hydroxyfriedelane (**23**)	CH_3_	CH_3_	H	CH_2_OH		[12,14,15,16]
**Pentacyclic triterpenes**		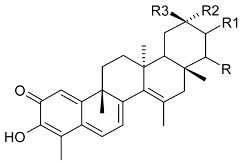					
		**R**	**R1**	**R2**	**R3**		
	scutione (**24**)	H	=O	H	CH_3_	Stem bark exudate	[15,18]
	netzahualcoyene (syn. vitideasin) (**25**)	H	H	COOCH_3_	CH_3_		[12,15]
**Pentacyclic triterpenes**		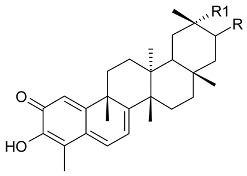					
		**R**		**R1**			
	pristimerin (**26**)	H		COOCH_3_		Stem bark exudate	[12,15,19]
	tingenone (**27**)	=O		H			[15,20]
	celastrol (**28**)	H		COOH			[12,15,21]
	epifriedelinol (syn. epifriedelanol) (**29**)	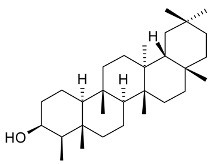				Leaves	[14,15]
	ilicifoline (D:A-friedoolean-1-en-29-ol-3-one) (**30**)	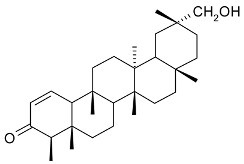				Stem bark exudates	[12,22]
	According to Torpocco (2007), it was isolated as olean-12-ene-3β,6β-diol. Considering given references, it was isolated as olean-12-ene-3β,16β-diol (syn. maniladiol, daturadiol) (**31**)	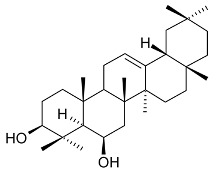				Stem bark exudates	[12,23]
	macrocarpine A (**32**)	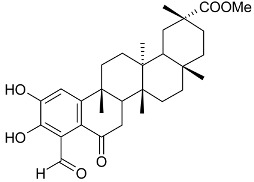				Root	[24]
	macrocarpine B (**33**) macrocarpine C (**34**)	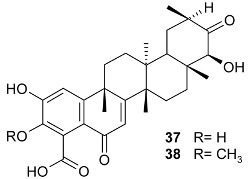				Root	[24]
	macrocarpine D (**35**)	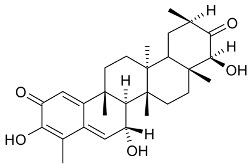				Root	[24]
				
		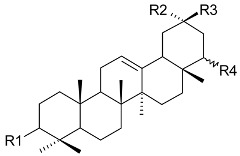					
		**R1**	**R2**	**R3**	**R4**		
	macrocarpoic acid A (3β, 22α-dihydroxy-olean-12-en-30-oic acid) (**36**)	β-OH	CH_3_	COOH	α-OH	Stem bark exudates	[13]
	macrocarpoic acid B (22α-hydroxy-olean-12-en-3-oxo-30-oic acid) (**37**)	=O	CH_3_	COOH	α-OH	Stem bark exudates	[13]
	maytenfolic acid (triptotriterpenic acid A, 3β,20α,22α-dihydroxy-olean-12-en-29-oic acid) (**38**)	β-OH	COOH	CH_3_	α-OH	Stem bark exudates	[13]
	triptotriterpenonic acid A (**39**)	=O	COOH	CH_3_	α-OH	Stem bark exudates	[13]
	22-*epi*-maytenfolic acid (triptotriterpenic acid B, 3β,22α-dihydroxy-olean-12-en-29-oic acid) (**40**)	β-OH	COOH	CH_3_	β-OH	Stem bark exudates	[13]
	22-*epi*-triptotriterpenonic acid A (**41**)	=O	COOH	CH_3_	β-OH	Stem bark exudates	[13]
	orthosphenic acid (**42**)	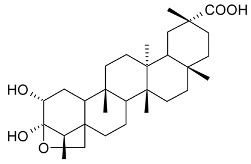				Stem bark extract	[13]
	3-(*E*)-coumaroyluvaol (macrocarpol A) (**43**) 3-(*E*)-caffeoyluvaol (**44**)	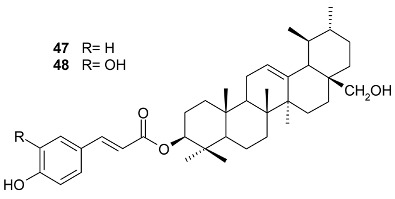				Stem bark extract	[13]
	octa-*nor*-13-hydroxydammara-1-en-3,17-dione (**45**)	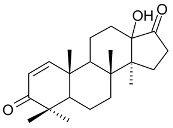				Stem bark extract	[12]
